# Tigecycline-Based Regimens for Complicated Urinary Tract Infections Caused by Carbapenem-Resistant Gram-Negative Bacteria: Case Series

**DOI:** 10.7759/cureus.65617

**Published:** 2024-07-29

**Authors:** Cristian-Mihail Niculae, Maria-Evelina Gorea, Laura-Georgiana Tirlescu, Raluca-Mihaela Matoru, Adriana Hristea

**Affiliations:** 1 Infectious Diseases, National Institute for Infectious Diseases “Prof. Dr. Matei Bals”, Bucharest, ROU; 2 Infectious Diseases, Faculty of Medicine, University of Medicine and Pharmacy “Carol Davila”, Bucharest, ROU

**Keywords:** acinetobacter baumannii, klebsiella pneumoniae, carbapenem resistant gram-negatives, bacteremia, urinary tract infections, tigecycline

## Abstract

There is existing controversy regarding the efficacy of tigecycline (TG) in treating complicated urinary tract infections (cUTIs) because of its pharmacokinetic concerns. We present three patients with cUTIs caused by carbapenem-resistant gram-negative (GN) pathogens successfully treated with high-dose tigecycline (HDT)-based regimens, as cefiderocol and aztreonam were not available in our country. The first case describes a 67-year-old patient with diabetes, prostate cancer, and double J ureteral stenting who was hospitalized with a febrile, complicated urinary tract infection (cUTI). Urine and blood cultures were positive for metallo-beta-lactamases (MBL)-producing extensively drug-resistant (XDR) *Klebsiella pneumoniae* (cefiderocol-susceptible). The synergy between TG and colistin using the in vitro E-test was demonstrated, and the patient was started on this regimen using HDT. Clinical and microbiological cures were achieved, and the patient was discharged home. The second case presents a 70-year-old patient with urethral pathology who was hospitalized with the diagnosis of a lower cUTI caused by an MBL-producing pan-drug-resistant (PDR) *Klebsiella pneumoniae*. The in vitro E-test showed synergy between TG and colistin, and our patient was successfully treated with this HDT-based combination. The third case emphasizes a 63-year-old patient with insulin-dependent diabetes, Child B cirrhosis, and a right double J ureteral stent who was hospitalized with a febrile cUTI. Urine and blood cultures were positive for carbapenem-resistant XDR *Acinetobacter baumannii* (susceptible to colistin and TG). Colistin was administered for only 96 hours because of stage II acute kidney injury, and we continued the treatment with HDT in monotherapy. The patient was discharged home, and no urinary tract infection relapse was seen for six months. Favorable clinical and microbiological outcomes were achieved with TG-based regimens in our cUTI cases. We highlight the role of antibiotic synergy determined by the *in vitro* E-test in two cases of MBL-producing XDR/PDR *Klebsiella pneumoniae*.

## Introduction

Tigecycline (TG) is a broad-spectrum antibiotic approved for treating community-acquired pneumonia, complicated intra-abdominal infections, and skin and soft tissue infections [1]. In addition, it is one of the last-resort drugs to treat carbapenem-resistant gram-negative (GN) expressing metallo-beta-lactamases (MBL) [2]. TG overcomes the main tetracycline resistance mechanisms, efflux pumps, and ribosomal protection [2]. However, TG-resistant bacterial mutants are not common as they come with high fitness costs [3]. The role of TG for treating complicated urinary tract infections (cUTIs) is not well defined in clinical practice because of the limited urinary recovery of active drugs and, consequently, a decreased concentration of active drugs at the site of infection [4]. According to international guidelines, complicated urinary tract infection (cUTI) was indicated by male sex, systemic symptoms (fever, chills, rigors), flank pain, tenderness in the costovertebral angle, indwelling urinary catheters or other devices, obstructive uropathy, any functional or anatomical abnormality with voiding disturbance, and/or systemic immunosuppression.

We present three cases of cUTIs caused by carbapenem-resistant GN successfully treated with high-dose TG-based regimens (HDT, 200 mg IV load, then 100 mg IV q12 h), as first-line treatment options were not available in our country and are still not available in most of the hospitals.

## Case presentation

Case 1

A 67-year-old male patient with a history of type II diabetes, prostate adenocarcinoma, treated with surgery and radiotherapy, chronic radiation-induced cystitis, bilateral double J ureteral stent, bilateral renal lithiasis, and a history of recurrent cUTIs with *Klebsiella pneumoniae* was hospitalized with a cUTI associated with fever. The patient reported a one-week onset of symptoms consisting of fever, dysuria, macroscopic hematuria, and right flank pain. On clinical examination, the patient was febrile, hemodynamically stable, and had right flank and lower abdomen pain. Right costovertebral angle tenderness was also noted. Laboratory tests revealed mild anemia, a high neutrophil count, elevated fibrinogen and C-reactive protein (CRP), and urinalysis revealed large leukocyturia, hematuria, and proteinuria without detection of nitrites (Table 1). Urine and blood cultures were performed using an automated microbial detection system, and empirical antibiotic treatment was initiated according to the patient’s medical history and the available drugs. The patient was started on the combination of colistin (9 million international units (MIU) loading dose), then 3 MIU q8h, and ceftazidime-avibactam 2.5 mg q8h, with no significant improvement over the following four days. Although the patient continued to be febrile, no signs of sepsis or hemodynamic instability were noted. Both urine and blood cultures were positive for MBL-producing *Klebsiella pneumoniae* expressing cefotaxime-M beta-lactamases (CTX-M), oxacillinase-48 carbapenemases (OXA-48), and New Delhi metallo-beta-lactamases (NDM) (Table 2). At that time, neither cefiderocol nor aztreonam were available in our country. The synergy between TG and colistin was demonstrated using the in vitro E-test. The patient was treated with a course of HDT and colistin for 14 days and has been afebrile since day five, along with a decrease in neutrophil count and inflammatory markers. After the confirmation of negative urine and blood cultures, the patient was referred to the urology department for replacement of the double-J stent. Since the beginning of treatment, the regimen has been hardly tolerated. The patient complained of digestive symptoms such as nausea, vomiting, and mild diffuse abdominal pain. The symptoms were successfully treated with supportive care. After 14 days, an acute pancreatic reaction with upper abdominal pain and an elevated lipase level of 741 U/L (reference range: 3-60 U/L) were noted. Abdominal ultrasound and CT scans ruled out acute pancreatitis; the patient was carefully monitored, and resolution occurred after the completion of antibiotic treatment. 

**Table 1:** Blood tests and urinalysis at hospital admission

Relevant blood tests and urinalysis	Case 1	Case 2	Case 3	Reference ranges
Hemoglobin (g/dl)	10.7	11.3	8.3	13.0-17.0
Neutrophil count (/µL)	7820	5800	5730	1.8-8.8
Fibrinogen (mg/dl)	673	634	330	200-393
C-reactive protein (mg/l)	222	24.8	30	0-3.0
Serum creatinine (mg/dl)	1.1	1.0	1.6	0.6-1.2
Serum urea (mg/dl)	39	35	49	15-45
White blood cells-urine (/µL)	19254	9059	2662	1-40
Red blood cells-urine (/µL)	7234	2666	79	1-17
Proteinuria	large	middle	middle	normal
Nitrites-urine	negative	positive	negative	negative

**Table 2:** Positive urine and blood cultures with drug susceptibility testing, including carbapenem MICs and other therapeutic options for carbapenem-resistant GN Note: Urine culture is defined as positive when a bacterial growth of at least 100,000 colony-forming-units/mL is detected. MIC: minimum inhibitory concentration; XDR: extensively drug-resistant; PDR: pan-drug-resistant; CTX-M: cefotaxime-M beta-lactamase; OXA-48: oxacillin-48-like carbapenemase; NDM: New Delhi metallo-beta-lactamase; ESBL: extended-spectrum beta-lactamase; MBL: metallo-beta-lactamase; S: susceptible; R: resistant; N/A: susceptibility or synergy test not performed; *Enterobacteriaceae strains with zone inhibition diameter ≥ 23 mm were considered susceptible, while those with zone inhibition diameter < 23 mm were considered resistant according to the EUCAST breakpoints; **Enterobacteriaceae strains with an MIC ≤ 0.5 mg/l were considered susceptible according to the EUCAST breakpoints; *** Synergy between tigecycline and colistin was demonstrated by the positive result of the E-test.

Antibiotic susceptibility testing	Case 1 K. pneumoniae	Case 2 K. pneumoniae	Case 3 A. baumannii
Resistance phenotype	XDR (CTX-M, OXA-48, NDM)	PDR (ESBL, MBL)	XDR
Meropenem MIC (mg/l)	>32 (R)	≥32 (R)	≥16 (R)
Imipenem MIC (mg/l)	>4 (R)	>4 (R)	>16 (R)
Ampicillin/sulbactam MIC (mg/l)	>8/4 (R)	>8/4 (R)	>8/4 (R)
Ceftazidime/avibactam MIC (mg/l)	>8/4 (R)	>8/4 (R)	N/A
Cefiderocol (Disk diffusion)*	S	R	N/A
Aztreonam MIC (mg/l)	>16 (R)	>16 (R)	N/A
Colistin MIC (mg/l)	>4 (R)	>4 (R)	≤0.5 (S)
Tigecycline MIC (mg/l)**	≤0.5	1	≤0.5
Fosfomycin MIC (mg/l)	>32 (R)	>32 (R)	>32 (R)
Gentamicin MIC (mg/l)	>4 (R)	>4 (R)	≥16 (R)
Amikacin MIC (mg/l)	>16 (R)	>16 (R)	>64 (R)
Tigecycline/Colistin synergy (E-test)***	positive	positive	N/A

Case 2

A 70-year-old male patient with a past medical history of an operated bulbar urethral stricture with secondary augmentation cystoplasty and an indwelling urinary catheter was admitted with the diagnosis of recurrent lower cUTI with multidrug-resistant (MDR) *Klebsiella pneumoniae*. The symptoms consisted of dysuria, pollakiuria, macroscopic hematuria, and lower abdominal pain 10 days before hospitalization. No fever was mentioned. On clinical examination, the patient was afebrile, hemodynamically stable, and had no abdominal or lumbar tenderness. Laboratory tests showed mild anemia and elevated inflammatory markers; urinalysis showed large leukocyturia and hematuria; moderate proteinuria; and a positive test for nitrites (Table 1). Urine and blood cultures were taken, and empirical antibiotic therapy with ceftazidime-avibactam 2.5 mg q8h and fosfomycin 8 g q8h was started based on the susceptibility testing from the previous episode. We received a confirmation of a positive urine culture with pan-drug-resistant (PDR) *Klebsiella pneumoniae* (Table 2). The in vitro E-test showed synergy between TG and colistin, and the patient was treated with HDT and colistin at a 9 MIU loading dose, then 3 MIU q8h for seven days. A negative urine culture was obtained at the end of the treatment along with favorable clinical and biological results, and the patient was referred to the urology department for catheter replacement. During treatment, the patient experienced nausea, loss of appetite, diffuse mild abdominal pain, and dizziness, with normal laboratory tests and remission under symptomatic treatment. 

Case 3

A 63-year-old male patient presented with a history of a one-week fever and right flank pain. The patient has a medical history of insulin-dependent diabetes, Child B cirrhosis, a right double J ureteral stent, and a previous episode of emphysematous pyelonephritis caused by a non-MDR *Escherichia coli*, complicated with urosepsis, purulent endophthalmitis, and left eye evisceration. Upon admission, the patient was hemodynamically stable and afebrile. Right-costovertebral angle tenderness was noted on clinical examination. Laboratory tests showed moderate anemia, elevated CRP, elevated serum creatinine, and elevated urea. Urinalysis showed leukocyturia, mild hematuria, moderate proteinuria, and a negative test for nitrites (Table 1). A native CT scan of the abdomen was performed, and no abnormalities were detected. Urine and blood cultures were positive for extensively drug-resistant (XDR) *Acinetobacter baumannii* (Table 2), and the patient was started on a colistin 6 MIU loading dose adjusted for renal function, then a 3.5 MIU q12h and HDT regimen. The ureteral stent was removed on day three. After 96 hours, the patient developed stage II acute kidney injury with a serum creatinine of 3.7 mg/dl, which required the discontinuation of colistin, and HDT monotherapy was continued as a salvage monotherapy regimen for up to seven days. During the treatment, the patient complained of severe digestive adverse effects. The treatment was stopped because of increasing liver enzymes, with aspartate aminotransferase (AST) of 248 U/L, reference range 14-36 U/L, and alanine aminotransferase (ALT) of 131 U/L, reference range 4-35 U/L, and mild coagulation disorder with hypofibrinogenemia (190 mg/dl, reference range 200-393 mg/dl), but without bleeding signs. The patient was discharged after the confirmation of negative urine and blood cultures, and the resolution of adverse effects, and no relapse of the urinary tract infection was observed for six months. 

## Discussion

There is limited data regarding the antibiotic treatment with TG in patients with cUTIs, especially when bacteremia is present. Traditionally, the use of TG for urinary tract infections (UTIs) has not been advocated because of its pharmacokinetic concerns [5]. The excretion of unchanged drug varies between 10% and 22% in different publications [5,6], with the expected concentration of drug in urine ranging from 7.5-11 mg/L or higher [7]. The average urine concentration of TG is expected to be several times higher than the minimum inhibitory concentration (MIC) for GN isolates found to be susceptible. However, monotherapy is generally not recommended in the presence of bacteremia [8]. Systematic reviews and publications based predominantly on case reports of patients with urinary tract infections with MDR *Klebsiella pneumoniae* or *Acinetobacter baumannii* concluded that TG appeared to have achieved some favorable clinical and microbiological results [6,9]. Some of them reported therapeutic success as measured by clinical cure and overall positive results ranging from 77.4% to 88.9% at a standard dose of 100 mg followed by 50 mg twice daily [5,10]. However, at this dose, bacteriuria may persist in about 50% of patients after completion of treatment [11]. Other systematic reviews have reported that HDT has been administered in patients with UTIs caused by *Klebsiella pneumoniae* in several reports, and microbiologic clearance has been achieved [10], so it may be a more reasonable treatment option for UTIs caused by MDR bacteria if effective source control is achieved. Current guidelines support the use of HDT only when there are no other options or as part of combination therapy [4]. Along with existing reports, our series supports the idea that TG-based therapies are a suitable option for the management of difficult-to-treat GN infections when no other options are available. For cUTIs associated with bacteremia, favorable short-term results can be achieved. However, when a device is used, there is a higher risk of infection relapse. For this purpose, we emphasize the importance of replacing the urinary catheter as soon as possible in accordance with existing guidelines [12,13]. Also, it is worth mentioning that in our cases, HDT was associated with colistin, except in one case in which colistin was discontinued due to severe side effects and TG was administered as monotherapy. The synergy between these two antibiotics is based on a mechanism in which colistin disrupts the outer and inner membranes of bacteria by destabilizing lipopolysaccharides [14], thereby increasing the permeability for other drugs [15], so that TG can easily enter the bacterial cell and bind to the 30 ribosomal subunit, inhibiting protein translation [1]. In some studies, the combination of these two antibiotics shows synergistic effects in about 70-80% of carbapenem-resistant *Klebsiella pneumoniae* strains [16,17]. Therefore, we emphasize the need to perform synergy testing, either by time-kill studies, e-tests, or microdilutions, in order to make the right treatment decision.

In terms of tolerability and safety, TG is known to cause a wide range of adverse effects. Digestive disorders such as nausea (28.5%), vomiting (19.4%), and diarrhea (11.6%) were the most frequent [18]. It is important to note that all the patients in our series experienced at least one adverse event, all of them complaining about digestive symptoms. However, none of them required discontinuation of therapy despite close monitoring, and all events were resolved at the end of treatment. Among the adverse events encountered, pancreatitis, liver damage, coagulopathy, and hypoglycemia are the most concerning, given the risk of an unfavorable outcome. The incidence of TG-induced pancreatitis is known to be less than 1%, although some publications suggest that the actual incidence may be much higher, around 20% [19]. The pancreatic reaction that occurred in our first case was characterized by diffuse abdominal pain and elevated lipase levels and was resolved spontaneously. The patient presented in our third case developed mild coagulopathy manifested by mild hypofibrinogenemia, decreased prothrombin time activity, and prothrombin time prolongation without bleeding signs. Routine monitoring of coagulation parameters is essential for patients receiving TG-based regimens. Some literature data suggest that attention should be paid to below-average fibrinogen levels, as the risk of bleeding and severe reactions is higher at fibrinogen levels below 1 g/L [20]. Regarding liver damage, TG has been reported to cause mild, transient increases in serum aminotransferase levels in 2 to 5% of recipients. However, in a cohort study of 35 patients, there was a 51.4% incidence of liver injury during treatment with the HDT regimen. Our patient developed a mild liver injury, expressed by elevated liver enzyme levels and anicteric cholestasis, which resolved after the TG discontinuation. At this point, we emphasize the need for further studies to investigate certain serious side effects, especially in HDT regimens, that should not be neglected during treatment, as the literature data on frequency and outcomes are currently very heterogeneous.

## Conclusions

TG is a salvage antibiotic that is currently not approved for the treatment of cUTIs, especially those associated with bacteremia, because of the lack of randomized control trials and limited data demonstrating efficacy. However, despite its associated adverse effects, it is one of the last options for the treatment of carbapenem-resistant gram-negative, especially when first-line therapies are not available. Our limited data suggest that in certain situations we can achieve successful results with clinical and microbiologic cures with HDT-based regimens in cUTIs. Also, we highlight the role of antibiotic synergy determined by the in vitro E-test in two cases of MBL-producing XDR/PDR *Klebsiella pneumoniae*. Well-designed clinical trials are needed to draw relevant conclusions regarding the use of tigecycline in cUTIs in order to avoid potential publication bias and produce good evidence-based recommendations.
